# Time-series dataset on land surface temperature, vegetation, built up areas and other climatic factors in top 20 global cities (2000–2018)

**DOI:** 10.1016/j.dib.2019.103803

**Published:** 2019-03-12

**Authors:** Yashar Jamei, Priyadarsini Rajagopalan, Qian Chayn Sun

**Affiliations:** a School of Property, Construction and Project Management, RMIT University, Melbourne, Australia; b Department of Geospatial Science, School of Science, RMIT University, Melbourne, Australia

**Keywords:** Google Earth Engine (GEE), LST, NDVI, NDBI, Time-series analysis, Top global cities

## Abstract

Time-series datasets of Land Surface Temperature (LST), Normalized Difference Vegetation Index (NDVI), Normalized Difference Built Index (NDBI) and other climatic factors are of significance due to their application in tracking climate change in cities. In this paper, new data processing methods are presented using the application of Google Earth Engine (GEE) and GIS. Different variables including LST (both daytime and nighttime), NDVI, NDBI, rainfall, wind speed, evapotranspiration, and surface soil moisture were computed for 18 years from 2000 to 2018 with of use of GEE platform. The study areas cover 20 top global cities which were mentioned in the global cities index report in 2018 [1]. The data sources used on GEE are: MODIS Terra LST and Emissivity 8-Day Global 1km; MODIS Terra Vegetation Indices 16-Day Global 1km; MODIS Terra Surface Reflectance 8-Day Global 500 m; TRMM Monthly Precipitation Estimate data; Terra Monthly Climate; MODIS Terra Net Evapotranspiration 8-Day Global 500 m; and NASA-USDA SMAP Global Soil Moisture Data. Also, to gather information regarding the global cities, United Nations (UN) population dataset, cities elevation and the A.T.Kerney report [1] was used. A short description of GEE functions to retrieve variables is provided. The dataset can be used to investigate the spatial-temporal relationships between LST, vegetation and built-up areas, as well as to provide the global perspective of climate and population change in various cities around the world.

**Table:** Specifications Table

Subject area	*Climate Change, Urban Planning*
More specific subject area	*Applied Remote Sensing and population growth*
Type of data	*Table and spatial dataset*
How data was acquired	*Acquired from multiple satellite images and open sources datasets*
Data format	*Derived Data, Analyzed Data with the final format of excel file and shapefile*
Experimental factors	*Satellite image processing, population data collection from UN and local city databases, and elevation values of cities from open sources*
Experimental features	*Combined the value of satellite derived data with population and elevation of each city and locate them in GIS*
Data source location	*Global cities retrieved from A.T.Kerney*[1]*report*
Data accessibility	*Data are available within this article*
Related research article	[2]

**Table:** 

**Value of the Data** • Presented dataset including day and night time LST, NDVI, and NDBI, other climatic factors, population and elevation in global cities from year 2000–2018 provide comprehensive information of climate change with respect to rising temperature over space and time. • Presented dataset can be utilized for statistical comparison of LST, rainfall, NDVI, NDBI and other variables and their trends between top 20 global cities. • This dataset is more beneficial for climate study compared to many other datasets which focused more on specific location (a single city), such as our previous work for Melbourne, Australia [3]. It can still be used to investigate the driving forces of climate change in individual cities.

## 1 Data

Derived GEE data of LST (both daytime and nighttime) [4], NDVI [5], NDBI [6], rainfall [7], wind speed [8], evapotranspiration [9] and surface soil moisture [10] were calculated from year 2000–2018 for top 20 global cities mentioned in 2018 Global Cities Report [1]. The sources of above-mentioned variables contain: MODIS Terra LST and Emissivity 8- L3 Global 1 km SIN Grid V006, MODIS Terra Vegetation Indices 16-Day L3 Global 1 km SIN Grid V006, MODIS Terra Surface 8-Day L3 Global 500 m SIN Grid V006, TRMM Monthly Precipitation Estimates data, Terra Monthly Climate and Climatic Water Balance for Global Terrestrial Surfaces, MODIS Terra Net Evapotranspiration 8-Day L4 Global 500 m SIN Grid V006, NASA-USDA SMAP Global Soil Moisture Data, UN population dataset [11], local city population and elevation datasets.

## 2 Experimental design, materials, and methods

**Fig. 1:**
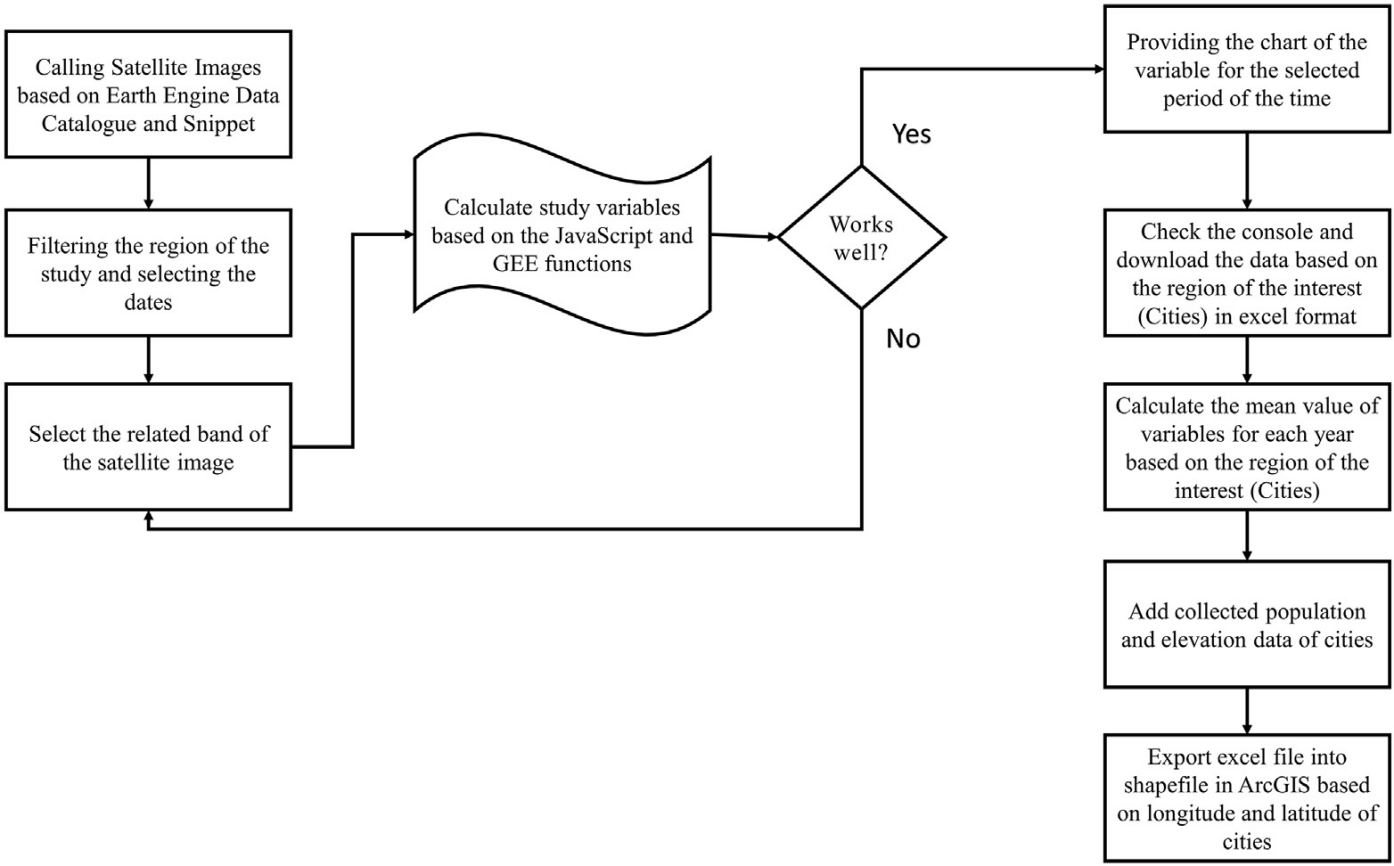
Methodological workflow for data processing using GEE and GIS.

Various satellite images were processed in the code editor with JavaScript in Google Earth Engine (GEE) to prepare the dataset of this research. First, the mean pixel value of the source satellite images in each city from year 2000–2018 were computed on GEE platform. Based on the temporal and spatial resolution of the satellite images, the raw dataset of the research variables was collated for each city from 2000 to 2018 in an excel file. The average value of each variable for each year was then calculated in the excel file. Next, we retrieved population and elevation data from UN and local city databases. Finally, based on the longitude and latitude of each city, the excel file was converted to shapefile format in ArcGIS software to create a spatial dataset. Fig. 1 shows the methodological work flow used in making this database.
