# Supplementary material for: Time-series dataset on land surface temperature, vegetation, built up areas and other climatic factors in top 20 global cities (2000–2018)
Source: Data Brief. 2019 Mar 12;23:103803. doi: 10.1016/j.dib.2019.103803 (PMC6660608; doi:10.1016/j.dib.2019.103803)
Supplement: Supplementary file 1 — Multimedia component 1 [file mmc1.pdf]

Conflicts of Interest Statement

Manuscript title: Time-Series data set on Land surface temperature, vegetation, built up areas and other climatic factors in Top 20 global cities (2000-2018)

The authors whose names are listed immediately below certify that they have NO affiliations with or involvement in any organization or entity with any financial interest (such as honoraria; educational grants; participation in speakers' bureaus; membership, employment, consultancies, stock ownership, or other equity interest; and expert testimony or patent-licensing arrangements), or non-financial interest (such as personal or professional relationships, affiliations, knowledge or beliefs) in the subject matter or materials discussed in this manuscript.

Author names:

Yashar Jamei

Priyadarsini Rajagopalan

Qian (Chayn) Sun

The authors whose names are listed immediately below report the following details of affiliation or involvement in an organization or entity with a financial or non-financial interest in the subject matter or materials discussed in this manuscript. Please specify the nature of the conflict on a separate sheet of paper if the space below is inadequate.

Author names:

This statement is signed by all the authors to indicate agreement that the above information is true and correct (*a photocopy of this form may be used if there are more than 10 authors*):

Author's name (typed)	Author's signature	Date
Yashar Jamei		07/02/2019
Qian (Chayn) Sun		07/02/2019
Priyadarsini Rajagopalan		08/02/2019
